# Transcriptome-based prediction of drugs, inhibiting cardiomyogenesis in human induced pluripotent stem cells

**DOI:** 10.1038/s41420-023-01616-6

**Published:** 2023-08-29

**Authors:** Anna Cherianidou, Franziska Kappenberg, Florian Seidel, Aviseka Acharya, Panagiota Papazoglou, Sureshkumar Perumal Srinivasan, Jürgen Hescheler, Luying Peng, Marcel Leist, Jan G. Hengstler, Jörg Rahnenführer, Agapios Sachinidis

**Affiliations:** 1 University of Cologne, Faculty of Medicine and University Hospital Cologne, Center for Physiology, Working Group Sachinidis, 50931 Cologne, Germany Robert-Koch-Str. 39, 50931 Cologne, Germany; 2 Department of Statistics, TU Dortmund University, Vogelpothsweg 87, 44227 Dortmund, Germany; 3 Leibniz Research Centre for Working Environment and Human Factors at the Technical University of Dortmund (IfADo), Ardeystrasse 67, 44139 Dortmund, Germany; 4 Heart Health Center, Shanghai East Hospital, School of Medicine, Tongji University, 200120, Shanghai and Research Units of Origin and Regulation of Heart Rhythm, Chinese Academy of Medical Sciences, 100730 Beijing, China; 5 In Vitro Toxicology and Biomedicine, Department of Biology, University of Konstanz, Universitätsstr. 10, PO Box M657, 78457 Constance, Germany

**Keywords:** Stem-cell differentiation, Stem-cell research

## Abstract

Animal studies for embryotoxicity evaluation of potential therapeutics and environmental factors are complex, costly, and time-consuming. Often, studies are not of human relevance because of species differences. In the present study, we recapitulated the process of cardiomyogenesis in human induced pluripotent stem cells (hiPSCs) by modulation of the Wnt signaling pathway to identify a key cardiomyogenesis gene signature that can be applied to identify compounds and/or stress factors compromising the cardiomyogenesis process. Among the 23 tested teratogens and 16 non-teratogens, we identified three retinoids including 13-*cis*-retinoic acid that completely block the process of cardiomyogenesis in hiPSCs. Moreover, we have identified an early gene signature consisting of 31 genes and associated biological processes that are severely affected by the retinoids. To predict the inhibitory potential of teratogens and non-teratogens in the process of cardiomyogenesis we established the “Developmental Cardiotoxicity Index” (CDI_31g_) that accurately differentiates teratogens and non-teratogens to do or do not affect the differentiation of hiPSCs to functional cardiomyocytes.

## Introduction

Human induced pluripotent stem cells (hiPSCs) offer the possibility of an unlimited cellular source for recapitulation of the multilineage differentiation to cells of the three germ layers, ectoderm, mesoderm, endoderm and further to specialized somatic cells such as cardiomyocytes (CMs), hepatocytes and neuronal cells. The somatic cells can be used for the monitoring of adverse effects of potential drugs and other environmental stressors [1, 2]. Although the number of drug candidates and chemicals is significantly increasing at present, traditional testing of embryotoxicity involves extensive animal studies which are costly, time-consuming and often the acquired findings are not relevant to the human situation as demonstrated by the example of thalidomide (THD) [3]. To overcome these limitations immense efforts are made to develop novel in vitro testing systems [4–9] based on pluripotent stem cells (PSCs) including human embryonic stem cells (hESCs) and hiPSCs-based systems [1, 10–15], especially to accomplish the rules of the REACH initiative [16]. Embryotoxicity occurs due to the detrimental effects of medicinal drugs or other environmental factors that may result in teratogenicity. Embryotoxic substances are capable to pass the placental barrier, thereby causing malformations of different organs of the embryo. The latter process is called teratogenesis. Recently, significant progress was made to develop in vitro test systems for identifying the teratogenic effects of drug candidates. We developed two test systems, which were based on hESCs and hiPSCs allowing quantification of the developmental toxicity potency of a compound based on wide DNA microarray transcriptome data [1, 14, 17, 18]. The so-called University of Konstanz 1 (UKN1) in vitro test system is based on neural induction of differentiation of hiPSCs towards the formation of neuroepithelial precursor cells (NEPs). In this context, UKN1 is well-established as in vitro test system for predicting of developmental neurotoxicity of several compounds [10, 19–21]. The first version of the Universitätsklinikum Köln (UKK1) test system partially recapitulates early embryonic development by random differentiation of hESCs or hiPSCs for 14 days under 3D embryoid body (EBs) conditions to three germ layers and their derivatives [11, 13, 22]. The test system was validated by exposing the PSCs (hESCs or hiPSCs) to various test compounds [10]. More recently, we developed a more efficient assay, the UKK2 test that can be applied under cell monolayer conditions and recapitulates early embryonic development by directed differentiation of hiPSCs to cells of all three germ layers by activating the Wnt signaling pathway [23]. We evaluated the UKN1 and UKK2 test systems in the presence and absence of 23 teratogens and 16 non-teratogens at the maximal plasma concentration (C_max_) and the 20-fold C_max_ concentration. After 6 days of neural induction (UKN1) or 24 h (germ layer induction) (UKK2) in the presence of teratogens and non-teratogens the total RNA was analyzed by whole genome-wide transcriptome microarrays. Based on the 1000 probe sets (PS) with highest variability across all samples and taking into consideration the cytotoxic effects of various compounds, we established a classifier which enables predicting the teratogens by both UKN1 and UKK2 test systems with a high accuracy of 87–90% and 90–92%, respectively [24, 25]. In addition, a combination of both test systems increased the predictive accuracy to 92–95% [24, 25]. The classification at 20-fold C_max_ resulted in similar accuracies by both test systems. To develop a developmental cardiotoxicity test system allowing discriminating teratogens and non-teratogens, we have identified an early specific cardiomyogenic gene signature that is essential for cardiomyogenesis in hiPSCs. In conclusion, we demonstrated that this in vitro model can be applied for predicting teratogens specifically affecting cardiac development.

## Results

### Directed differentiation of hiPSCs (SBAD2) towards cardiomyocytes after exposure to teratogens and non-teratogens

**Fig. 1:**
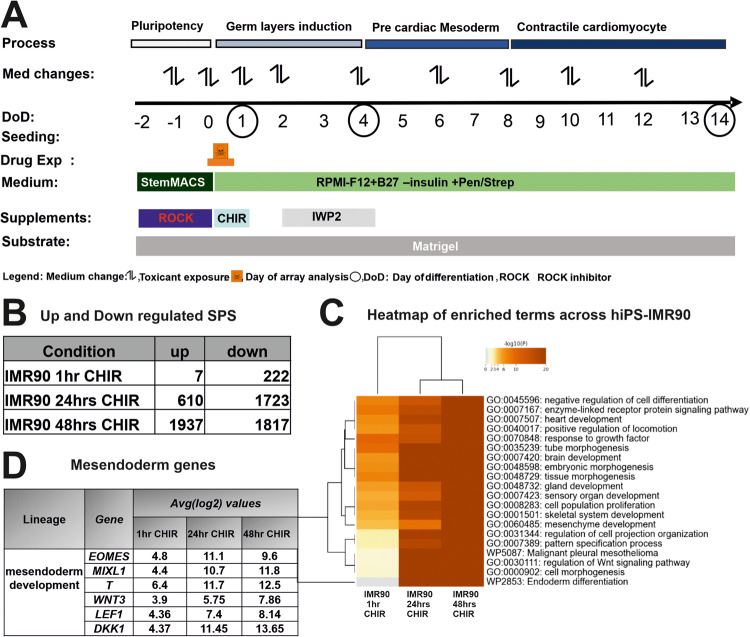
Transcriptome analysis of differentiated hiPSCs (IMR90) toward germ layer cells. **A** Overview of the UKK2-CTT for the in vitro cardiac differentiation of the experimental design from day −2 to day14. **B** To determine the the early germ layer formation, hiPSCs were exposure to 1 hr, 24 h (day1) and 48 h CHIR (day2) compared with those of untreated controls. The hiPSCs were cultured as a monolayer on matrigel-coated plates for 2 days under pluripotent conditions and on day 0 exposed to GSK3 inhibitor, CHIR (10 µM) for 24 h. After microarray analysis of the RNA, the number of the up- and down regulated SPSs (log2 fold change > 1; adjusted *p*‐value < 0.05) were determined. **C** Visualization of enriched gene ontology terms across IMR90 1 h, 24 h and 48 h after CHIR. Heatmap showing the top 20 enrichment clusters, colored by *p*-values. **D** Mesendoderm gene table Representative mesendoderm genes increased gradually for the different time points, after exposure to CHIR in IMR90-hiPSCs.

**Fig. 2:**
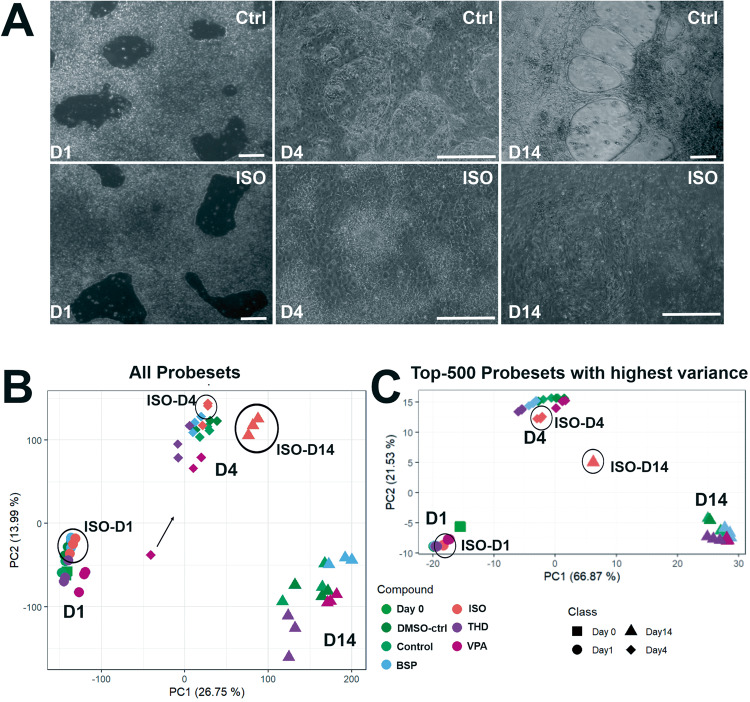
Transcriptome analysis of differentiated hiPSCs (IMR90) toward CMs. The hiPSCs were cultured as a monolayer on matrigel-coated plates for 2 days under pluripotent conditions and on day 0 exposed to GSK3 inhibitor, CHIR (10 µM) for 24 h. After 48 h exposed to Wnt inhibitor, IWP2 (5 µM). Spontaneously beating cardiac clusters were observed from day 9 onwards. Simultaneously, cells were exposed to test substances for a single exposure of 24 h (day1). The cells were harvested for gene array analysis on day1, day4 and day14 (Fig. 1A). Medium changes were done as indicated every alternate date. **A** Representative phase-contrast images of control and ISO treated hiPSC at day1-, 4 -and 14day. Scale bar, 100 µm. **B** PCA blot of 54,675 probe sets for three timepoints during the differentiation. **C** PCA blot of the 500 SPS with the highest variance across the mean of the condition‐wise samples. The respective day is indicated by the shape and the respective measured compound is indicated by the color of the dot, as labels are shown next to the plots. The distribution of the data points on the x-axis is given by the PC 1 and on the y-axis by PC2. The percentages in parentheses denote the proportion of explained variance for the respective PC.

To study the early events of the process of cardiomyogenesis, we used a cell monolayer-based directed hiPSC differentiation protocol, designated as the UKK2 cardiotoxicity test (UKK2-CTT), which is based on the sequential activation and inhibition of Wnt/β-catenin signaling [23] (Fig. 1A). The serial induction of differentiation with WNT signaling, with the small molecule Wnt/β-catenin agonist CHIR, leads to a transition from pluripotency (day0) to the three germ layers as demonstrated by our transcriptome findings at the end of day2 (Fig. 1B). As we previously described, using the hiPSCs (SBAD2 origin) we could discriminate almost all 23 teratogens (including 13-cis-retinoic acid (ISO)) from the 16 non-teratogens based on their transcriptomes at the end of day1 [24]. The teratogens and non-teratogens were applied in two concentrations, the plasma peak concentration (C_max_) and the 20-fold C_max_ concentration [24]. According to the cardiomyogenic UKK2-CTT, CHIR was removed after 24 h incubation for the next 24 h (day2) and then IWP2 (Wnt/β-catenin small molecule inhibitor) was added to the medium for the following 48 h (day4) to facilitate the transition from mesodermal cells to cardiac progenitors at day4 of differentiation. At day4, aggregated forms of the cells began to emerge in the entire monolayer culture forming a network of branches. The contractile activity was observed at only random spots on day 8, whereas the entire cell monolayer network of cells was synchronously beating on day14 (Fig. 2A). The purity of cardiomyocytes at day14 was higher than >90 % by this differentiation protocol [2].

Interestingly, among all teratogens and non-teratogens the teratogens (Table 1) ISO, 9-cis-retinoic acid (RA) and Acitretin that are known to act through the retinoid receptors (RR), completely inhibited the cardiomyogenesis process, since no beating cluster and no spontaneous beating areas could be identified and cardiac sarcomere was absent.

**Table 1:** Compounds, abbreviations, C_max_ concentrations, beating profile and CDI score for non teratogens and teratogens.

Compound	Abbreviation	Tested concentration [µM]	Beating^a^	CDI score
		1-fold C_max_^b^		
Non-teratogens				
Ampicillin	AMP	107	Yes	0.1^c^
Ascorbic acid	ASC	200	Yes	0
Buspirone	BSP	0.0244	Yes	0
Chlorpheniramine	CPA	0.0304	Yes	0
Dextromethorphan	DEX	0.15	Yes	0
Diphenhydramine	DPH	0.3	Yes	0
Doxylamine	DOA	0.38	Yes	0.03
Famotidine	FAM	1.06	Yes	0
Folic acid	FOA	0.38	Yes	0.03
Levothyroxine	LEV	0.077	Yes	0.03
Liothyronine	LIO	0.00307	Yes	0.06^d^
Magnesium (chloride)	MAG	1200	Yes	0
Methicillin	MET	140	Yes	0
Ranitidine	RAN	0.8	Yes	0
Retinol	RET	1	Yes	0
Sucralose	SUC	2.5	Yes	0.2
Teratogens				
9-cis-Retinoic acid	9RA	1	No	1
Acitretin	ACI	1.2	No	1
Isotretinoin	ISO	1.7	No	1
Atorvastatin	ATO	0.54	Yes	0
Carbamazepine	CMZ	19	Yes	0.03
Entinostat	ENT	0.2	Yes	0.2^e^
Favipiravir	FPV	382	Yes	0
Leflunomide	LFL	370	Yes	0.3^f^
Lithium (chloride)	LTH	1000	Yes	0.2^g^
Methotrexate	MTX	1	Yes	0.2
Methylmercury	MEM	0.02	Yes	0.1^h^
Paroxetine	PAX	1.2	Yes	0.2^i^
Teriflunomide	TER	370	Yes	0.3^j^
Thalidomide	THD	3.9	Yes	0.3
Trichostatin A	TSA	0.01	Yes	0.1^k^
Valproic acid	VPA	600	Yes	0.4
Vismodegib	VIS	20	Yes	0

^a^Yes, if beating was observed; No, if beating was not observed.

^b^Υes^,^ if on day 14 was observed beating cardiomyocytes; No, if on day 14 were not observed any beating cardiomyocytes. The CDI score is defined as Cardiotoxicity Developmental Index. This index has a maximal value of 1, which is reached when all 31 genes from (Fig. 5E) are deregulated by a compound; likewise, it has a minimal value of 0 if no gene is deregulated. The CDI score is calculated only on the hiPSC-SBAD2 cells. The beating and cytoxocity refers to hiPSC-IMR90 as well as hiPSC-SBAD2 indicate a different deregulation pattern as the retinoinds and the 31 ‘’gold standard” genes.

^c^Total 3 out 31, 2 downregulated instead of up regulated like in retinoids.

^d^Total 7 out 31, 1 downregulated instead of up regulated like in retinoids.

^e^Total 6 out 31, 2 downregulated instead of up regulated like in retinoids, 3 upregulated instead of being downregulated like in retinoinds.

^f^Total 10 out 31, 4 downregulated instead of up regulated like in retinoids, 1 is upregulated instead of being downregulated like in retinoinds.

^g^Total 7 out 31, 5 downregulated instead of up regulated like in retinoids, 1 is upregulated instead of being downregulated like in retinoinds.

^h^Total 4 out 31, 3 downregulated instead of up regulated like in retinoids, 1 is upregulated instead of being downregulated like in retinoinds.

^i^Total 6 out 31, 1 downregulated instead of up regulated like in retinoids.

^j^Total 8 out 31, 3 downregulated instead of up regulated like in retinoids.

^k^Total 4 out 31, 1 downregulated instead of up regulated like in retinoids, 1 is upregulated instead of being downregulated like in retinoinds.

### Directed differentiation of hiPSCs (IMR90 origin) towards cardiomyocytes after exposure to isotretinoin, thalidomide and valproic acid

To further discriminate the impact of RR activation for the teratogens specifically inhibiting cardiomyogenesis in differentiating IMR90 hiPSCs, four test compounds were chosen, among them ISO (as a gold standard in our study), Valproic acid (VPA) and Thalidomide (THD) as well as Buspirone (BSP) as a non-teratogen. To identify the optimal time for early germ layer formation induced by CHIR through activation of the canonical Wnt/β-catenin signaling pathway to mesoderm and further to cardiomyocytes, we compared the transcriptomes at 1 h, 24 h (day1) and 48 h (day2). The hiPSCs were cultured in the presence of CHIR for 1 h, 1 day (day1) and then CHIR and cells were cultured for further 24 h in the absence of CHIR (day2). The number of the differentially expressed genes (significant probe sets, SPS) increased proportionally with the incubation time (Fig. 1B). In the presence of CHIR for 24 h (day1), 610 SPS were significantly up and 1723 SPS were significantly downregulated. GO analysis of the DEGs (Fig. 1C) indicated that several developmental pathways such as: -embryonic morphogenesis-, - heart development (mesodermal origin), -brain development (ectodermal origin and partial gland development (endodermal origin) were significantly enriched at these time points. This observation was confirmed by the strong deregulation of mesoderm genes such as *T* (Brachyury), *EOMES*, and *MIXL1*, three classical and conserved mesodermal factors that control the exit of pluripotency and germ layer segregation [26]. *WNT3*, *LEF1* and *DKK1*, markers for initiating the process of cardiomyogenesis, were also highly upregulated (Fig. 1D, table). These findings indicate the high activation of several differentiation processes that recapitulate embryonic development. Moreover, the findings suggest that the initiation of germ layer formation occurred at day1 of differentiation. Therefore, to identify the cardiomyogenic pathways in hiPSCs, we performed transcriptomic analysis after differentiation of the hiPSCs for 1, 4 and 14 days in the presence and absence of the three selected teratogens (ISO, VPA, THD) and one non-teratogen (BSP). Among them, ISO has been shown as a clear inhibitor of cardiomyogenesis in hiPSCs (Fig. 2A, control and ISO morphology). RNA samples were harvested at day1 (initiation of germ layers), day4 (early cardiac progenitor cells) and day14 (CMs). As shown in Fig. 2A, in contrast to the control cardiomyocytes (day14), which indicated beating clusters of cardiomyocytes, day14 differentiated ISO-treated hiPSCs showed a static morphology without any beating clusters of cardiomyocytes. To obtain an overview of genome-wide gene expression alterations induced by the different test compounds, principal component analysis (PCA) was performed based on all 54,675 analyzed probe sets (Fig. 2B) and based on the top 500 with the highest variance (Fig. 2C). The principal components PC1 and PC2 explain the shown percentages (%) of the variance of the transcriptomes at the different differentiation periods (Fig. 2B, C). The three replicates for each time point clustered relatively closely together suggesting a good reproducibility of the microarray data. The transcriptomes of the ISO-14-day triplicate were the only ones that exhibited a noticeable separation from the control, VPA-, THD-, and BSP-14-day triplicate transcriptomes in the PC1 direction, indicating that ISO had an impact on cardiomyogenesis. (Fig. 2B). A clear separation of the ISO-14-days transcriptome in both PCA directions was observed taking in consideration the top 500 SPS with the highest variance (Fig. 2C).

**Fig. 3:**
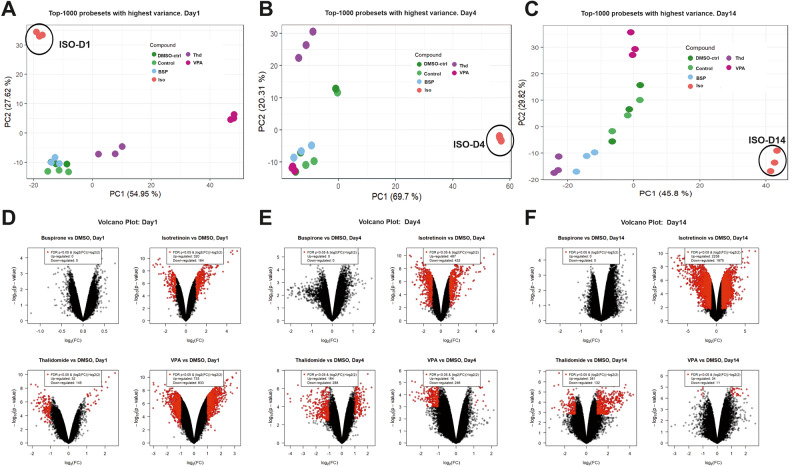
Principal component analysis (PCA) & Volcano plots of deregulated probe sets of teratogenic and non-teratogenic compounds. Comparison of the top1000 SPS with highest variance for ISO-, THD- and VPA-treated vs control day1, day4, and day14 differentiated hiPSCs (IMR90). **A**–**C** PCA-plots for each day, respectively. The different compounds are indicated by different colors. The distribution of the data points on the x-axis is given by the PC 1 and on the y-axis by PC2. The percentages in parentheses denote the proportion of explained variance for the respective PC. **D**–**F** Volcano plots of deregulated SPS for ISO-, THD- and VPA-treated vs control day1, day4, and day14 differentiated hiPSCs. Each dot represents one out of 54,675 probe sets from the Affymetrix gene chips. The fold‐ change of the differentially expressed probe sets in substance‐exposed cells is given on the x‐axis in log2‐values, and the corresponding *p*‐values of the limma‐analyses are given on the y‐axis in negative log10‐values. Red dots represent SPS with a statistically significant, FDR‐adjusted *p*‐value < 0.05 and an absolute fold‐change > 2. The numbers of up‐ and downregulated red‐dot‐probe sets are indicated.

To compare the differences between the 3 teratogens (ISO, VPA, THD) and the non-teratogen BSP at different periods of differentiation, we performed a new PCA with the 1000 SPS with the highest variance (Fig. 3A–C) for each differentiation time point. As indicated the day1, day4 and day14 transcriptomes of BSP cluster together with the appropriate transcriptomes of the control (without DMSO) and DMSO-control (end concentration 0.1%). Interestingly, a clear separation of the transcriptomes of the three teratogens (ISO, VPA and THD) was observed at all three differentiation time points (Fig. 3A–C). Genome‐wide expression changes were also illustrated in volcano plots for a comparison of selected test compounds day1, day4, day14 vs the appropriate controls (DMSO-control, day1, day4 and day14) respectively, (Fig. 3D–F), (at least 2‐fold deregulated; FDR *p*‐value < 0.05). In general, a large number of SPS was obtained for the teratogens, in particular for ISO, whereas none was observed for the non‐teratogen BSP. Interestingly, on day1 and day4, the two other teratogens, THD and VPA, showed also a high number of deregulated genes. Nevertheless, the number of developmental genes for ISO at day4 and day14 was much higher than the genes deregulated by VPA and THD, correlating with the complete inhibition of cardiomyogenesis by ISO.

### Identification of differentiation processes at day1 of hiPSCs (IMR90) differentiation affected by isothretinoin, valproic acid, thalidomide and buspirone

**Fig. 4:**
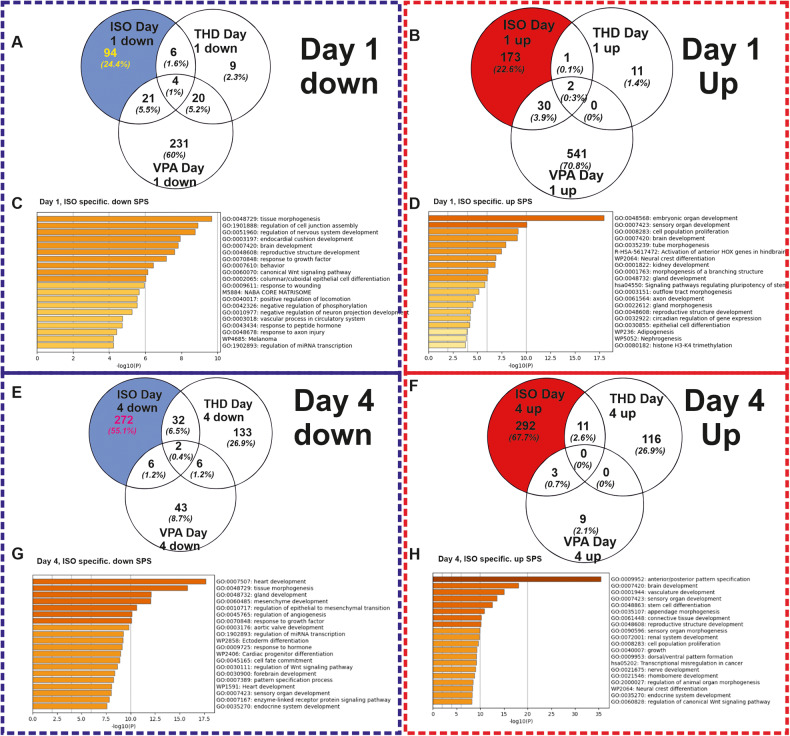
Biological interpretation of the ISO-specific differentially expressed genes after exposure of hiPSCs (IMR90) to ISO, THD and VPA at day1. **A**, **B** The Venn diagrams shows the number of down-regulated and up-regulated SPS (log2 fold change > 1; adjusted *p*‐value < 0.05), respectively induced by the selected compounds. **C**, **D** Metascape analysis for specific ISO-induced downregulated and upregulated SPS, respectively. Analysis shows the statistically enriched BPs and pathways as colored by the *p* values. **E**, **F** The Venn diagrams shows the number of down-regulated and up-regulated SPS (log2 fold change > 1; adjusted *p*‐value < 0.05), respectively induced by the selected compounds. **G**, **H** Metascape analysis for specific ISO-induced downregulated and upregulated SPS, respectively. Analysis shows the statistically enriched BPs and pathways as colored by the *p* values.

To study the biological significance of the differentially expressed genes, we compared the SPS (FDR *p*-value < 0.05; log2 fold change ≥ 2), of ISO when compared to others (VPA, THD and BSP), during the transition through mesoderm at day1. The ISO-specific exposure led to 273 (94 downregulated and 173 upregulated) SPS (FDR *p*-value < 0.05; log 2fold change ≥ 2) (Fig. 4A, B). To characterize the biological functions of genes deregulated by the three teratogens at day1, the up-and downregulated SPS were separately analyzed by the Metascape functional enrichment tool https://metascape.org/gp/index.html#/main/step1) [27]. No deregulated SPS were identified by BSP as a non-teratogen.

The GO analysis of the ISO-specific downregulated genes recognized enriched GOs such as -tissue morphogenesis-, -regulation of nervous system development-,- endocardial cushion development -and the -canonical Wnt pathway signaling- (Fig, 4C). The corresponding genes belonging to these GOs are shown in Supplementary Fig. S1A (Supplementary Fig. S1A). The KEGG analysis reveals pathways such as Ras and PI3k-Akt Signaling (Supplementary Fig. S1B). Analysis of the ISO-specific upregulated SPS recognized prominent general early developmental GOs, such as -embryonic organ development-, -brain development-, -activation of anterior HOX genes in hindbrain, -neural crest differentiation- (Fig. 4D). The table with the GO genes (Supplementary Fig. S1C) reveals the genes that are related to the above-mentioned significant processes and KEGG pathways (Supplementary Fig. S1D), such as -signaling pathways regulating pluripotency-, -TGF-beta signaling- (all having a crucial role during general developmental processes).

The most prominent enriched GOs with their particular genes of the downregulated (Supplementary Fig. S1A) and upregulated genes (Supplementary Fig. S1C) are presented, respectively. As anticipated, critical genes necessary for heart development, such as BMP2, DKK1, EOMES, and GATA4, were identified within the canonical Wnt signaling pathway. Notably, the expression of these genes involved in heart development was downregulated following ISO-day1 treatment. Analysis of the upregulated genes at ISO-day1 (Supplementary Fig. S1C) resulted in the identification of general developmental GOs such as - pattern specification processes, -heart development-, and -brain development- (genes of the specific GOs are shown in Supplementary Fig. S1C table). The analysis of the top 50 genes specifically down- and upregulated by ISO (Supplementary Fig. S2A, B) revealed significant GO terms associated with the observed effects. These findings suggest a suppression of cardiomyogenesis and the initiation of neurogenesis as prominent biological processes influenced by ISO treatment. As indicated the Wnt signaling that normally is activated by CHIR in DMSO-control is downregulated in ISO-treated hiPSCs. Validation of the microarray data at day1 was performed with five arbitrarily selected genes by qPCR. The qPCR data analysis confirmed the deregulation pattern of these genes (in square with star, see Supplementary Fig. S2C) and underlined the significant deregulation of ISO-treated conditions when compared to the other three compounds.

### Identification of differentiation processes at day4 of hiPSCs (IMR90) differentiation affected by Isotretinoin, VPA, Thalidomide and Buspirone

A similar analysis was performed by analyzing the ISO-specific deregulated genes at day4, which recapitulates the transition from cardiac mesoderm differentiating towards cardiac progenitors. ISO-specific set exposure led to 574 (272 downregulated and 292 upregulated) SPS (FDR *p*-value < 0.05; log2 fold change ≥ 2) (Fig. 4E, F). The downregulated genes were classified to GOs mainly associated with mesoderm-derived organs such as -heart development-, and ectoderm-derived organs such as -brain development- (Fig. 4G). The table with the GO genes (Supplementary Fig. S3A) shows the genes of the heart-related downregulated enriched terms and the KEGG pathways such as -TGF-beta signaling-, -dilated cardiomyopathy- and-hypertrophic myopathy- (Supplementary Fig. S3B).

The upregulated genes are enriched in GOs that mainly are involved in the -anterior posterior patter specification- processes, -brain development and other early morphogenesis-developmental processes (Fig. 4H) additional the table with the GO genes (Supplementary Fig. S3C) and KEGG pathways associated with Hedgehog-, Hippo-, Wnt and pluripotency regulating signaling pathways (Supplementary Fig. S3D). The top 50 ISO-specific upregulated (Supplementary Fig. S4A) genes belong to GOs which are crucial for pattern specification processes whereas the downregulated (Supplementary Fig. S4B) are involved in signaling pathways inducing heart development. Validation of the microarray data at day4 was done by the expression of 7 arbitrary genes using qPCR. The qPCR data analysis confirmed the deregulation pattern of these genes (Supplementary Fig. S4C).

Taken together, the analysis of ISO specific set on day1 and day4, revealed that genes represented by the GO terms -heart Development-, and signaling pathways including Wnt/β-catenin, TGF beta, BMP signaling, which are all known to regulate mesodermal differentiation, are potential biomarker candidates for a successful transition towards mesodermal state and play a key role for fate specification in the heart development.

The significantly deregulated VPA-specific genes at day1 and the THD-specific deregulated genes at day4 were also analyzed by Metascape. Heart development GOs are provided in the Supplementary Fig. S5 (Supplementary Fig. S5A–K). Since at day14 we got beating clusters of cardiomyocytes we may conclude that VPA and THD have no significant effects on mesoderm-depending cardiomyogenesis but may partially inhibit the development of functionally intact cardiomyocytes.

### Identification of a shared pattern after Retinoid exposure between two different hiPSC lines

**Fig. 5:**
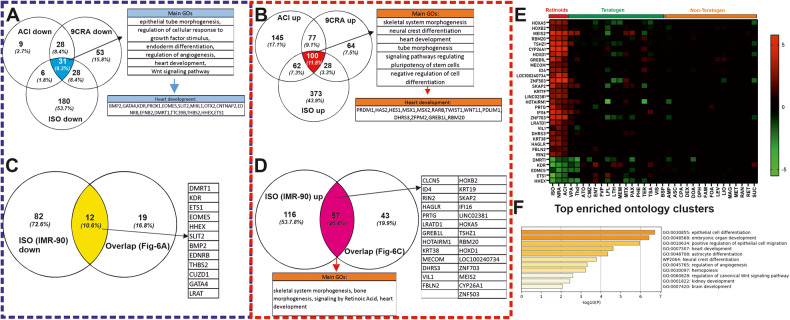
Common gene signature between the differential expressed genes at day1 in ISO-treated SPDA2 hiPSCs and ISO-treated IMR90 hiPSCs. **A**, **B** Venn diagrams show the number of common retinoids specific (ISO; 9-cis-retinoic acid: 9CRA and Acitretin: ACI) downregulated and upregulated genes, respectively, in SBAD2 hiPSC at day1. The commonly down- and upregulated genes (31 and 100 respectively) were analyzed by the Metascape tool to identify the statistically enriched GOs and pathways. **C**, **D** The Venn diagrams show the number of the common genes between the ISO-specific upregulated and downregulated genes at day1 in IMR90 hiPSCs, respectively, and in the Retinoids specific genes in SBAD2 as shown in **A** and **B** commonly down- and upregulated genes: 12 and 57 respectively). **E** The heatmap shows the 31 down- and upregulated genes out of the 69 differential expressed common genes (12 and 57) with a log2FC value higher than 1. On the x-axis are the abbreviations of the teratogens and non-teratogens (Table 1 in ref. [24]) and on the y-axis the 31 commonly deregulated genes. **F** Metascape analysis of the 31 selected genes showing prominent enriched BPs and pathways colored by *p*-values.

Next, we compared the transcriptomes of the 3 retinoids (Acitrecin, 9CRA and ISO) in the SBAD2 hiPSCs [24] with the transcriptome of ISO in IMR90 hiPSCs at day1 of differentiation (Fig. 5). In this context, the three retinoid compounds completely inhibited the cardiomyogenesis as observed at day14 when we differentiated the SBAD2 hiPSCs and the IMR90 hiPSCs in the presence of ISO.

The overlapping up- or downregulated SPS at day1 as compared to the control conditions, we identified 31 downregulated (Fig. 5A) and 100 upregulated (Fig. 5B) common genes between all three retinoids at day1 in the differentiating SBAD2 hiPSCs as compared to control cells after reanalyzing the day1 SBAD2 hiPSCs transcriptome data [24]. Further analysis across the common retinoid compounds SBAD2 hiPSCs day1 data and the IMR90 hiPSCs day1 ISO data resulted in 69 common genes, among them 12 downregulated (Fig. 5C) and 57 upregulated (Fig. 5D) between all three retinoids at day1 of differentiation.

Among these genes, we selected 31 genes of key biologically significant and highly deregulated (Fig. 5E) (at least 2fold up- or downregulated). The expression levels of these genes across all the teratogens and non-teratogens are shown in the heatmap (Fig. 5E). The heatmap indicates a clear separation of retinoid, teratogens and non-teratogens making these genes strong candidates for potential cardiac mesodermal markers, which are essential for cardiac development. The enriched GO analysis of these genes by Metascape revealed significant developmental processes, such as embryonic organ development, heart development, angiogenesis and regulation of WNT signaling pathway (Fig. 5F).

### Impact of teratogens and non-teratogens on beating activity of the cardiomyocytes

**Fig. 6:**
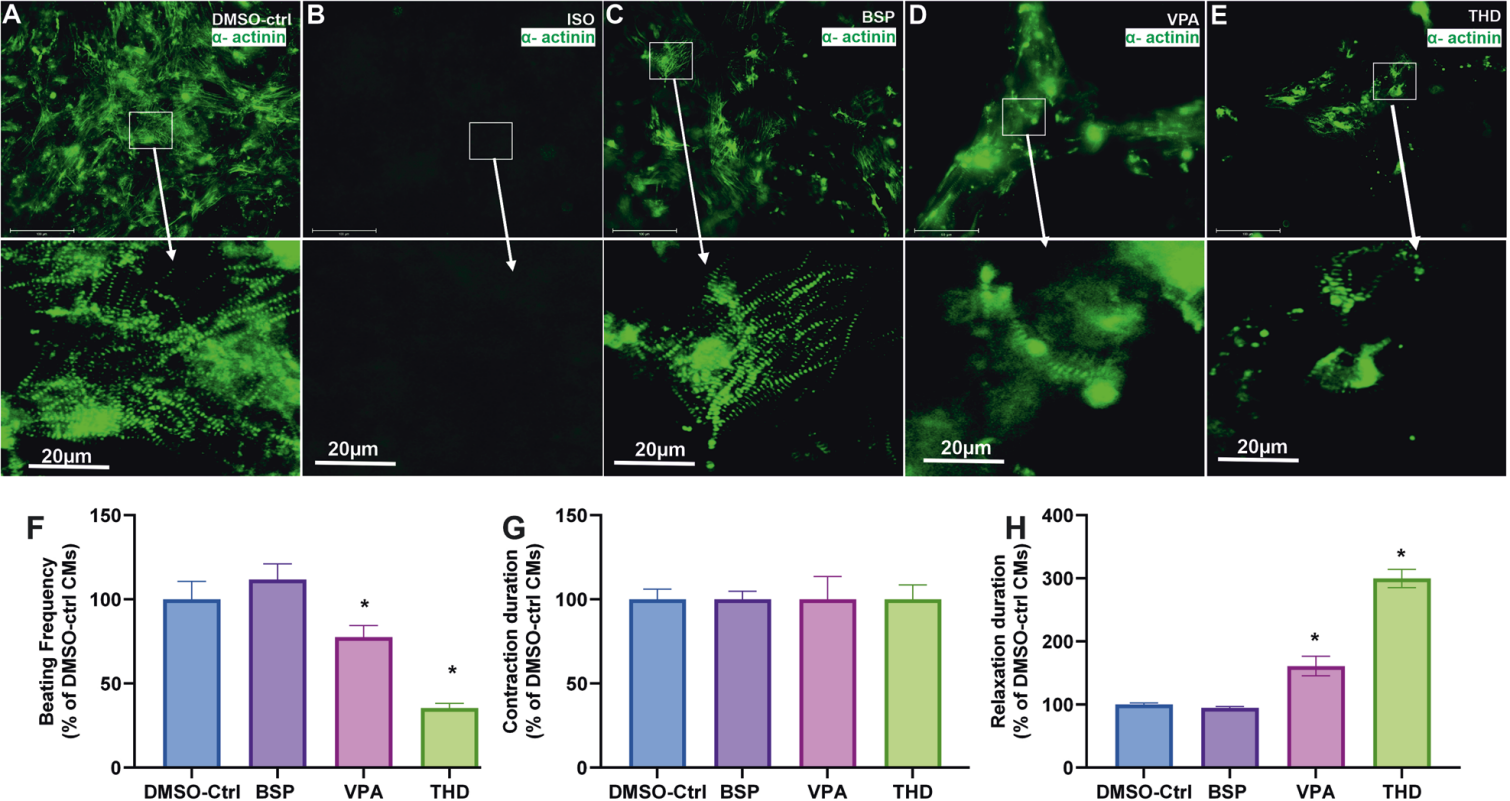
Effects of ISO, THD and VPA on contractility of ACTN2 copGFP^+^-CMs on day14. **A**–**C**, **E** Representative immunofluorescence live imaging of sarcomeric ACTN2-copGFP^+^-CMs at day14 obtained after differentiation of IMR90 ACTN2 copGFP^+^-hiPScCs on day14 in the absence and presence of ISO, VPA, BSP and THD, respectively (Scale bar: 100 μm (original) and 20 µm (magnified)), arrows indicate the sarcomere striation morphology. **F**–**H** The diagrams show the beating frequency, the contraction and the relaxation duration at day14, respectively, for control, BSB, VPA and THD. The values are expressed as a percentage of the control CMs values, which were set to 100%. (mean ± SEM, *n* = 3, *p < 0.05).

We further carried out a general assessment of cardiomyocyte differentiation until day14 based on the beating activity of the SBAD2 and IMR90 CMs as compared to the control CMs. Among all teratogens tested with the UKK2-CTT, only the retinoids completely inhibited the formation of beating cardiomyocytes at day14, whereas treatment with the non-teratogens had no effects on the beating frequency of the cardiomyocytes at day14 (Fig. 6A–F). To focus on well-documented teratogens such as VPA and THD on the cardiomyogenesis process we generated a live imaging transgenic IMR90 hiPSCs cell line using the CRISPR-Cas9 and a homology-directed recombination approach as we described previously [2, 28]. The ACTN2-cop green fluorescent protein (ACTN2-cop-eGFP^+^-hiPSC line (IMR90 origin) enables the live imaging of sarcomeres after differentiation to ACTN2-cop-eGFP^+^-CMs, since the α-cardiac specific actinin (ACTN2) is enriched in the sarcomeres, the smallest contractile unit of cardiomyocytes.

As illustrated in Fig. 6A the control day14 ACTN2-cop-eGFP^+^-CMs show an intact muscle striation structure and a beating activity of 65 beats per min (Supplementary Video S1). As expected, no cardiomyocytes were observed at day14 in the presence of ISO (Fig. 6B). The top 50 deregulated genes visualized in a heat map (Supplementary Fig. S6A) reflect the influence of ISO on the transcriptome on day14 and the absence of any cardiac markers. The non-teratogen BSP did neither affect the striation structure of the CMs nor the beating activity (Fig. 6C and Supplementary Video S2). However, VPA and THD compromised the cardiac muscle striation of ACTN2-cop-eGFP^+^-hiPSC (Fig. 6D, E, respectively) and reduced the beating activity as compared to control, BSP and other non-teratogens. Using the software VA1.9 [2, 28], we also analyzed the beating frequency and the fluctuations of the contraction and relaxation velocity of the differentiated cardiomyocytes on day14, as has already been described [2, 28]. As shown in Fig. 6F, VPA and THD exposed CMs resulted in decreased beating frequency when compared to control and the non-teratogen BSP. There was no significant change in the contraction velocity, in contrast to the relaxation phase that was drastically increased in VPA and almost 3 times more in THD (Fig. 6E, G).

In general, to identify a teratogen that specifically inhibits cardiac development based on the retinoids gene signature, we defined the “Cardiac Developmental Index” (CDI_31g_). This index has a maximal value of 1 for the retinoid compounds (=no of deregulated genes divided by 31 deregulated genes). In the case that no gene is differentially expressed the CDI_31g_ value for the compound has a minimum value of zero. Therefore, all three retinoids deregulated the expression of 31 developmental-related genes (5 down- and 26 up-regulated, in the SBAD2 cell line). When compared to the non-teratogens, the teratogens yielded in a higher CDI_31g_ score. The teratogens VPA and THD showed 12 and 10 deregulated genes, with CDI_31g_ scores of 0.38 and 0.32 respectively (Table 1). The distribution of these genes in IMR90 cell line (Supplementary Fig. S6B) confirmed their high deregulation when compared to the other two teratogens (VPA and THD) and the non-teratogen, BSP. The live cell imagining revealed the deterioration of the sarcomeric a-actinin and the irregular structure of the actinin filaments in VPA (Fig. 6D) and THD (Fig. 6E) treated conditions when compared to untreated cardiomyocytes. The non-teratogen BSP did not affect the beating activity and muscle striation. Accordingly, a CDI score of 0 was calculated.

## Discussion

In the present study, we imitated cardiomyogenesis in hiPSCs by applying the Wnt signaling dependent differentiation protocol of hiPSCs to recognize key cardiomyogenesis gene signatures that can be applied to identify compounds and/or other environmental factors specifically inhibiting the cardiomyogenesis process. For this aim, we developed the UKK2-CTT that is based on a published cardiomyocyte differentiation protocol [23] with small modifications in the presence and absence of teratogens and non-teratogen at previously reported plasma peak concentrations (C_max_) [24]. Interestingly, only the retinoids induced a complete inhibition of the process of cardiomyogenesis. Therefore, we extended our study by using the teratogens ISO, VPA and THD and the non-teratogen BSP to identify specific cardiomyogenic gene signature/pathways by performing transcriptome analysis at the different stages of differentiation, combined with testing of functional alterations during CMs contractions. It is well known that the teratogen THD causes malformations of the limbs [29] whereas VPA induces congenital malformations and is a neurological teratogen [30, 31].

The vertebrate heart is developed from lateral mesoderm during gastrulation [32, 33]. Normal mesoderm is formed when epiblast cells enter through the primitive streak (PS) during gastrulation, a process that requires the synchronized action of BMP, Nodal, Wnt, and FGF signaling pathways (reviewed in [34]). A Nodal- mediated suppression of neural development depends on active Wnt signaling as was reported previously [35]. Wnt/β-catenin signaling has a biphasic role in controlling the differentiation of cardiomyocytes in vivo and in scalable in vitro models. As demonstrated in the zebrafish and ESC model at early stages of development, Wnt/β-catenin signaling promotes cardiogenesis in embryonic stem cells, whereas, at later stages, it contributes to the proper size of the heart-forming field [33, 36].

It is well known that balanced RA signaling is involved in multiple stages of heart development including the formation of cardiac mesoderm and specification of cardiomyocytes to different cardiac cell subtypes [37]. During intact embryonic development, RA signaling is essential in the process of cardiogenesis. However, an unbalanced activation of the Retinoic signaling pathway is teratogenic for heart development in vertebrates [38]. Production of RA is tightly regulated by enzymes, which expression levels vary considerably during embryonic development [39]. Therefore, the timing and the RA concentration is crucial for the differentiation processes toward cardiomyogenesis. In addition, the concentration of RA and cardiomyogenic factors such as BMP2 differentiation of hiPSCs can be directed in specified cardiac cell types such as sinoatrial node or epicardial cardiomyocytes. It was also demonstrated that the addition of a high concentration of RA (0.5–1 mM) in mesoderm-differentiated hiPSCs induces different CMs cell types [37]. Relatively high RA concentrations also promote the development of neural progenitors in hESC EBs [40] via simultaneous inhibition of the Nodal/Activin and BMP signaling pathways [41]. In addition, previous studies have already demonstrated that RA represses mesodermal cell fates [42] and inhibits the RA signaling pathway resulting in a transient overexpression of T Brachyury and Mixl, two transcriptional factors required for mesoderm formation [43, 44].

In UKK2-CTT, the exposure of hiPSCs simultaneously to CHIR (a WNT activator) and retinoids (ISO, RA, Acitretin), resulted in enriched neuronal development events and inhibited mesoderm induction. The retinoid-treated hiPSCs indicated an increased expression of HOX genes in hindbrain during embryonic development and decreased the level of the transcription factor eomesodermin (EOMES), required for specification of the early stages of heart development and the direction of pre-cardiac mesoderm fate specification [45] via dysregulation of Wnt signaling molecules such as HHEX, ZNF503, and KDR. Our findings suggest that the inhibition of cardiogenic mesoderm specification due to Retinoids exposure could happen because of a shift in mesoderm patterning toward a more -anterior primitive streak phenotype.

The excessive RA signaling is accompanied by an increase of HOX activity during heart development in zebrafish embryos and results in the loss of both atrial and ventricular cardiomyocytes [46]. We also observed a strong upregulation of HOXA5, HOXB5 and HOXD1 RNA levels after RA treatment at day1 suggesting that the HOXA family plays a role in RA-induced heart teratogenicity.

Interestingly*, LEFTY1 and LEFTY2* (TGF-β superfamily members), which play an important role in cardiomyogenesis, were highly upregulated under retinoid-treated conditions starting from day1 (Supplementary Fig. S5L, M, respectively). Both factors play an important role in left and right pattering and their upregulation normally inhibits heart development via inhibition of NODAL signaling pathway [47, 48]. Our results are consistent with the observation that when mouse embryonal carcinoma cells differentiate with RA, there is an increase in Lefty expression [49]. The binding of both, LEFTY1 and LEFTY2 to Nodal inhibits the Nodal signaling pathway via the inactivation of the active Nodal/Activin receptor complex [50]. The last findings suggest that the loss of function of Nodal prevents mesoderm formation, which has an impact on further cardiac specification during embryonic development.

Based on two different hiPSC lines and three different retinoids we could identify an early heart developmental gene signature (31 genes) involved in heart development. We evaluated the predictive potency of the UKK2-CTT by defining the CDI_31g_ index for teratogens and non-teratogens (Table 1). As indicated, the CTX_31_ value for the teratogens had values around 0, indicative for non-teratogenicity. Interestingly the CTX_31_ values for THD and VPA was 0.3 and 0.4 respectively. The values suggest some teratogenic effects, especially for THD, for cardiac development. In this context, it was shown that indeed THD and VPA exerted some cardiac defects in developing chicken embryos [51] and mice [52], respectively. Moreover, THD treatment of patients with multiple myeloma (MM) induced cardiotoxicity in more than 50% of patients, which was manifested in severe bradycardia [53, 54]. Moreover, THD induced also severe bradycardia in patients suffering from amyotrophic lateral sclerosis (ALS) [54, 55]. We observed beating clusters of CMs in hiPSCs after treatment with THD and VPA for 24 hrours, indicating that these compounds did not significantly affect cardiomyogenesis in these cells. However, the beating frequency of the resulting cardiomyocytes was significantly reduced by both THD and VPA due to the prolongation of the relaxation phase, suggesting that the functional properties of the developed cardiomyocytes were affected. Interestingly, this CMs phenotype was observed in cancer and ALS patients who developed bradycardia after THD treatment (for review see ref. [54]).

Till now the mEST model is the only alternative test method based on the differentiation of murine ESCs (mESCs)/EBs model toward beating cardiomyocytes [56]. The mEST cardiotoxicity model was validated by the European Center for the Validation of Alternative Methods (ECVAM) and THD was classified as a weak embryotoxic agent. Moreover, mimicking the mEST model, it has been reported that THD inhibits cardiomyogenesis in the hiPSCs-EB model at an IC_50_ concentration of 117 µg/ml. The findings presented in the study were based on the EB model and a simple beating assay of CMs using a high concentration of THD (453 µM), which was 117-fold higher than the C_max_ concentration (3.9 µM) used in our study. As a result, the inhibition of cardiomyogenesis observed by the authors may be due to nonspecific cytotoxic effects. Our UKK-CTT method has several advantages, including the ability to study specific signaling pathways of cardiomyogenesis at the earliest stages of mesodermal formation, and it can be performed rapidly under monolayer conditions at C_max_ concentrations. This allows for the screening of multiple substances based on the CDI_31g_ index, as evaluated by non-teratogens. Additionally, our test can discriminate teratogens that specifically inhibit the process of functional cardiomyogenesis.

## Materials and methods

### Test compounds, teratogenicity information and Plasma peack concetrations

The test compounds were purchased from Sigma‐Aldrich (St. Louis, MO, USA). These were buspirone hydrochloride (B7148, CAS# 33386‐08‐2), isotretinoin (PHR1188, CAS# 4759‐48‐2), thalidomide (T144, CAS# 50‐35‐1), and valproic acid (PHR1061, CAS# 99‐66‐1). All compounds were dissolved and stored at 20,000‐fold C_max_ concentrations in 100% DMSO (Carl Roth, Germany) or, alternatively, in distilled water, if soluble. The tested concentrations (C_max_) of the teratogens and non-teratogens as well as the information on teratogenicity correspond to previously published studies [54, 55]. Briefly, the set of compounds were selected based on three criteria. A first inclusion criterion was the availability of published information on whether a compound is teratogenic or non-teratogenic in humans and/or animals. The second inclusion criterion was the availability of pharmacokinetic information from clinical studies and other resources, so that therapeutic compound concentrations (C_max_) could be calculated as concentrations for in vitro testing. A third inclusion criterion was sufficient solubility so that the C_max_ in culture medium could be achieved by maximally 0.5% DMSO as a solvent.

For UKK cardio differentiation, Thalidomide (T144), Valproic acid (PHR1061) and Isotretinoin (PHR1188) were chosen as representative teratogens and Buspirone (B7148) as a representative of a non-teratogen at 1 fold of the C_max_ concentration. All were purchased from Sigma Aldrich ((St. Louis, Missouri, USA). These compounds were solved and stored in concentrations of 20.000-fold C_max_ in 100% DMSO or alternatively in distilled water, if soluble.

### Human induced pluripotent stem cells

SBAD2 cells, a human induced pluripotent stem cell line was originally produced for the StemBANCC project (http://stembancc.org) and received from Prof. Marcel Leist (University of Konstanz). The Leibniz-Institute DSMZ (German Collection of Microorganisms and Cell Cultures) validated the cell identity by short tandem repeat profiling.

The IMR90 hiPSC (authorized by the Robert-Koch Institute; Berlin, Germany, license number: AZ 3.04.0210083) was used to generate the transgenic α-cardiac actinin (ACTN2)-copepod (cop) green fluorescent protein (GFP+)-human-induced pluripotent stem cell line by using the CRISPR-Cas9 and a homology directed recombination approach, as described in Acharya et al. [54, 55].

These cells were cultured and maintained in StemMACS™ iPS-Brew XF basal medium (Miltenyi Biotec, Germany) supplemented with 10 mL StemMACS iPS-Brew XF, 50X supplement, (Miltenyi Biotec, Germany) along with onto Matrigel-coated plates (Corning GmbH, Germany), as already been described [54, 55].

### Differentiation of hiPSCs towards germ layers and further to cardiomyocytes

hiPSC cells on pluripotent state, were dissociated with CTS™ TrypLE™ Select Enzyme (Thermo Fisher Scientific, Germany) and seeded at a densitiy of 600,000 cells per well on Matrigel coated 6-well-plates in StemMACS™ iPS-Brew XF medium, supplemented with 10 µM ROCK inhibitor Y-27632 (Calbiochem, Merck KGaA, Darmstadt, Germany). On the next day, the medium was changed to StemMACS™ iPS-Brew XF medium without adding ROCK inhibitor. On day 0, the differentiation was induced by adding 10 µM Wnt activator small molecule CHIR (R&D Systems, Minneapolis, USA) in RPMI 1640 GlutaMAX™ medium (Thermo Fisher Scientific, Germany) plus B-27™ Supplement, minus insulin (Thermo Fisher Scientific, Germany). At the same time, the cells were incubated (5% CO_2_, 37 °C) with the test compounds at a 1-fold C_max_ and a DMSO concentration of 0.1% as DMSO-control. The medium was then changed to basal RPMI/B-27-ins medium and cells were kept for further 24 h. At day 2, RPMI/B-27-ins medium with small molecule WNT inhibitor IWP2 (Tocris, United Kingdom) 5 µM was added and cells were kept for 48 h (day 2 to day4). Afterwards, cells were maintained in basal RPMI/B-27-ins media and spontaneously beating clusters were visible by day 9 onwards.

After CHIR exposure, the cells were collected for RNA extraction after 1 h, 24 h and 48 h (24 h CHIR exposure) and the RNA extraction for the compound treated conditions along with the corresponding controls (DMSO-controls) collected on day1, day4 and day14. For each tested condition, three biological replicates were generated.

### Video analyzer

To analyze the beating activity and the sarcomere contractive activity of the CMs on day14 we used the software Video Analyzer (1.9) as described in Acharya et al. [54, 55]. The videos and fluorescent live images ware taken using EVOS Cell Imaging Systems (CMMC, Cologne).

### RNA isolation

The TRIzol lysis reagent (Thermo Fisher Scientific, Germany) was used for the homogenization of the cells and the total RNA isolation was done using the RNeasy Mini Kit (Qiagen, Germany) according to the manufacturer’s instructions. Total RNA concentration was measure by UV-Vis spectrophotometer nanodrop2000c (Thermo Fisher, Germany). The samples were proceeded for microarray gene expression studies using kits, reagents and instruments from Affymetrix or for qRT-PCR validation.

### qRT-PCR

The mRNA expression analysis was executed as we described previously as described [54, 55]. In brief, 500 ng of mRNA was used to synthesize cDNA (SuperScript Vilo, Invitrogen). Then cDNA synthesis was diluted with highly pure RNAse free water at 1:5 ratio and then 2 μL of the diluted cDNA was used for qRT-PCR (Applied Biosystems 7500 FAST Real-Time PCR System) with the selected primers (Supplementary Table S1) and GAPDH mRNA was used as an internal control.

### Microarray labeling and hybridization

The global gene expression monitoring was conducted using the Affymetrix Microarray gene expression study and the protocol as described in [57]. In brief, 100 ng of total RNA was used to perform the microarray.

After amplification, the samples were labeled using GeneChip 3′ IVT Express Kit mRNA with biotin labeled as per the manufacturer’s protocol (Affymetrix, High Wycombe, UK). With the use of magnetic beads the samples were purified. After fragmentation of these, the samples were hybridized on Affymetrix Human Genome U133 Plus 2.0 arrays (Affymetrix, Santa Clara, CA, USA) for 16 h at 60 rpm and 45 °C. Post hybridization, the arrays were washed, stained and subjected to the scanning using Affymetrix GeneChip Scanner-3000-7G. The scanner generated ‘. CEL’ files which were used for further downstream analysis.

### Statistical methods

The analyses were conducted using the statistical software R, version 4.2.2, with additional R-packages as indicated in the following sections. For each combination of compound and day, three independent biological replicates were considered.

Along with the R, we also used TAC4.1 tool to process the CEL-files to get the gene list for the downstream analysis. The log2 fold change was kept as +/−2 as cutoff. These gene were processed via online tool such as Metascape to enrich GOs from the lists. Additional Venn diagrams were generated using the online platform VENNY 2.1.

### Data pre-processing

The Affymetrix CEL-files were pre-processed using the frozen robust multi-array average (fRMA) algorithm that consists of the three steps background correction, normalization, and summarization. This yields expression values for 54,675 probe sets (PS). For this, the software R, version 4.2.2 [25] and the R-packages affy [26], frma [27], and hgu133plus2frmavecs [28] were used.

### PCA plots

Principal component analyses (PCA) were based on the pre-processed expression values. Plots were based once on all 54675 PS and once only on the top 1000 or top 500 PS with respect to their variance across all considered samples. Both the scenarios with all samples (all compounds, all days) and with only the samples corresponding to the individual days were considered.

### Limma analysis

The R-package limma [29] was used for the calculation of differential expression between the samples treated with the different compounds and DMSO, per day, respectively. The limma (linear models for microarray data) approach is an empirical Bayes method, where the complete set of all PS was considered for the adjustment of the variance estimates of single PS. The resulting moderated t-test is abbreviated here as ‘limma t-test’. Resulting *p*-values were multiplicity adjusted to control the false discovery rate (FDR) by the Benjamini–Hochberg procedure [30]. The resulting gene list for each compound comprises estimates for the fold-change (FC), log2 fold-change, and the *p*-values of the limma t-test (unadjusted and FDR-adjusted), as well as the calculated mean values on original or on log2-scale for the DMSO samples and the respective compound. Results of the differential expression analyses are shown via Volcano plots, where on the x-axis, the log2 FC and on the y-axis, -log10 of the unadjusted p-value was plotted for each PS. PS with an adjusted p-value smaller than 0.05 and an absolute value of the log2 FC larger than log2(2) = 1 were considered to be a significant PS (SPS).

### Venn diagrams, top genes, GO group overrepresentation and KEGG pathway enrichment analyses

Venn diagrams were created for the comparison of sets of SPS for the test compounds, once based on all sets of SPS, once only for SPS that were upregulated and once for SPS that were downregulated. Here, only SPS with a gene annotation were used. For each Isotretinoin specific part of the Venn diagrams, lists of the corresponding top50 genes were determined. SPS were sorted according to their adjusted *p*-values, and the top 50 SPS with a gene annotation were chosen out of all SPS, and for the up- and downregulated SPS separately. Normalized expression values of these topgenes were displayed using heatmaps. Additionally, the SPS significant only for Isotretinoin for each day (i.e. the Isotretinoin specific part of the Venn diagrams) individually were analyzed with respect to enriched GO groups and KEGG pathways. This was done both for up- and downregulated SPS.

For the KEGG pathway analysis, the SPS were assigned to their respective KEGG pathway. Using Fisher’s exact test, it was statistically tested whether more PS assigned to the specific pathway were differentially expressed than expected at random. KEGG pathway analyses were conducted using the R package clusterProfiler.

### Statistical analysis

If not otherwise indicated in the text, then analysis was performed using a one-way pairwise ANOVA test or t-test and *p* values < 0.05 were considered statistically significant.

### Supplementary information

Figure S1

Figure S2

Figure S3

Figure S4

Figure S5

Figure S6

Table S1

Video S1

Video S2

## Data Availability

The microarray data have been deposited in the Gene Expression Omnibus (GEO) (NCBI): GSE187001; GSE233924 and GSE233926.
