# Supplementary figures and images for: Transcriptome-based prediction of drugs, inhibiting cardiomyogenesis in human induced pluripotent stem cells
Source: Cell Death Discov. 2023 Aug 29;9:321. doi: 10.1038/s41420-023-01616-6 (PMC10465524; doi:10.1038/s41420-023-01616-6)

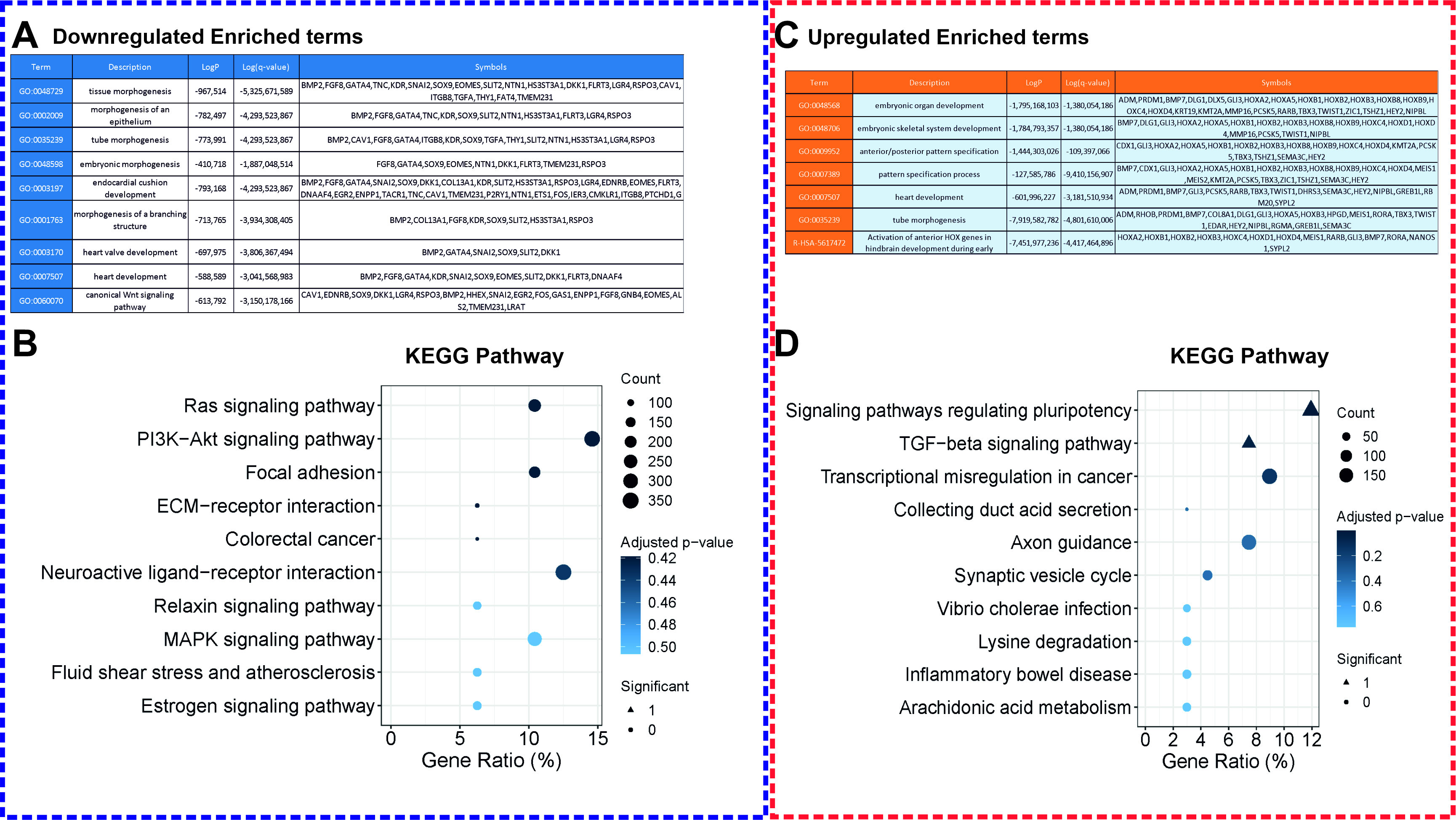

Supplement: Supplementary file 1 — Figure S1 [file 41420_2023_1616_MOESM1_ESM.jpg]

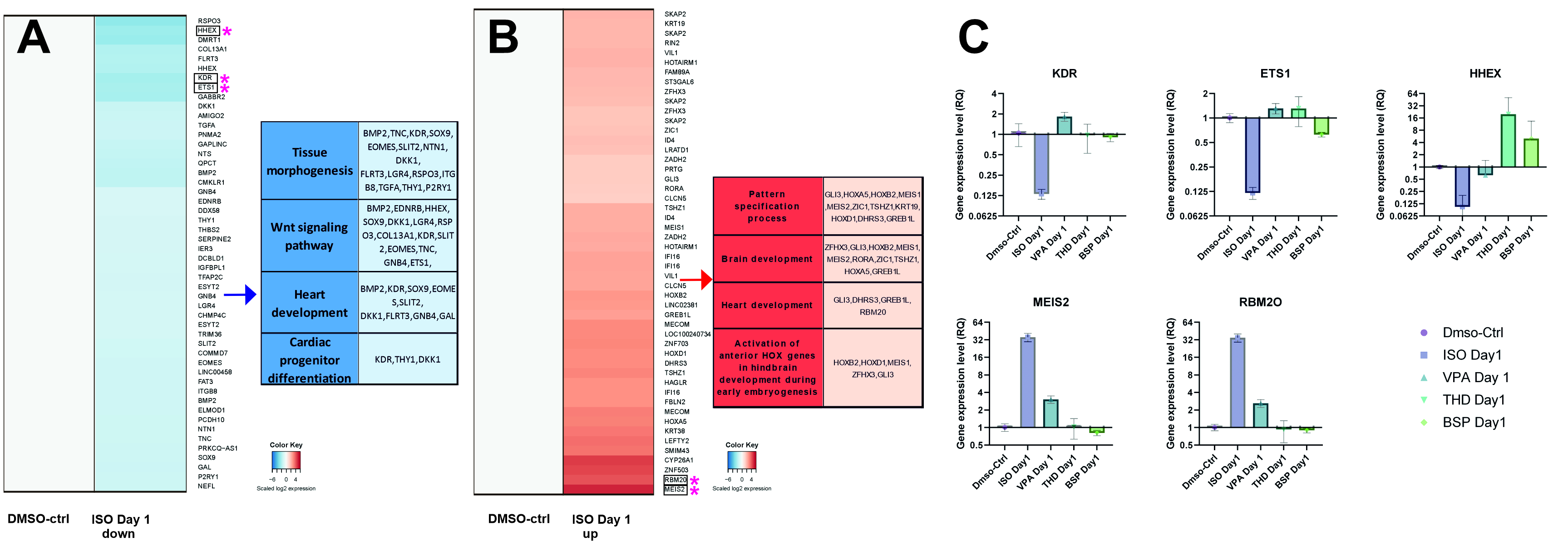

Supplement: Supplementary file 2 — Figure S2 [file 41420_2023_1616_MOESM2_ESM.jpg]

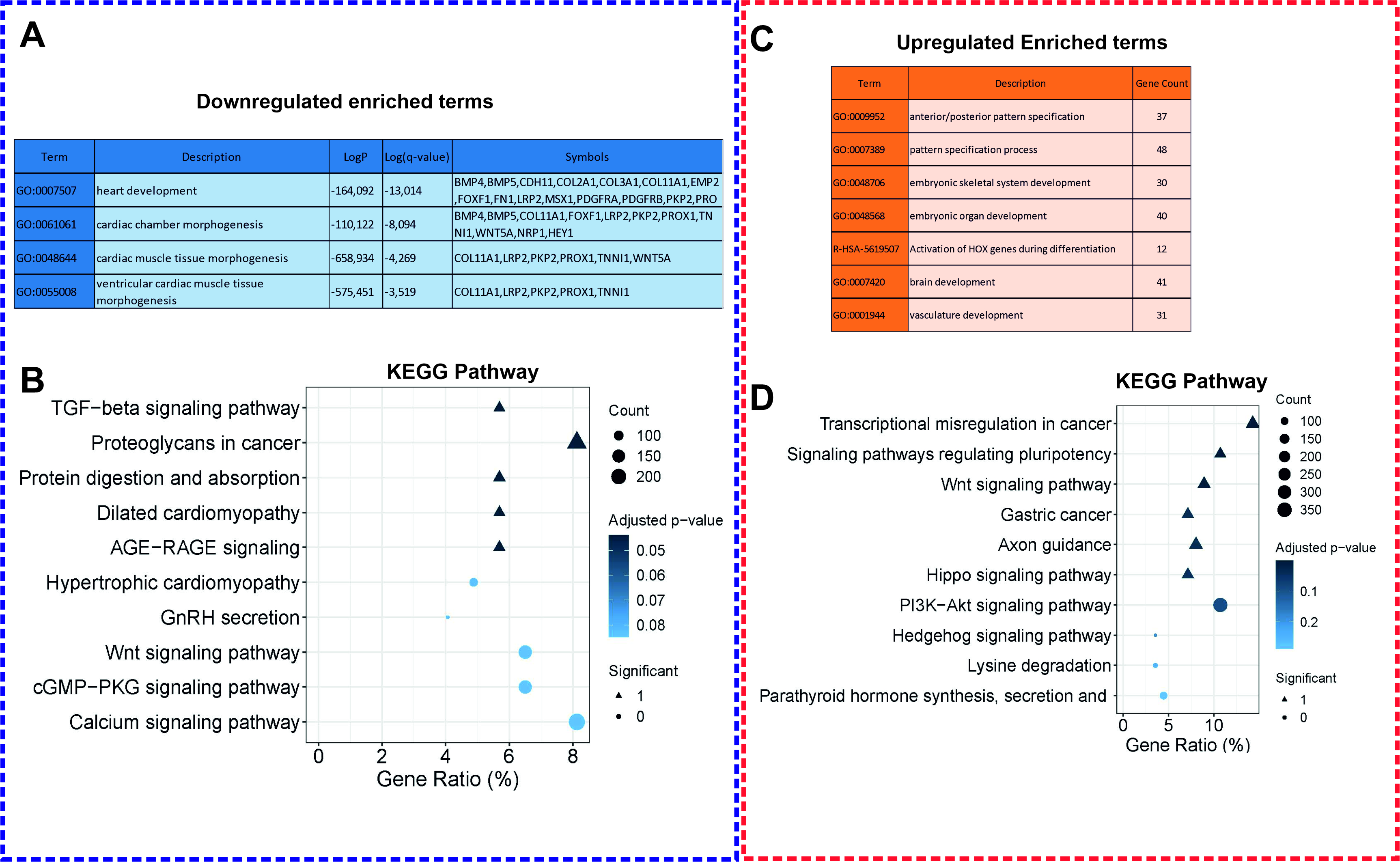

Supplement: Supplementary file 3 — Figure S3 [file 41420_2023_1616_MOESM3_ESM.jpg]

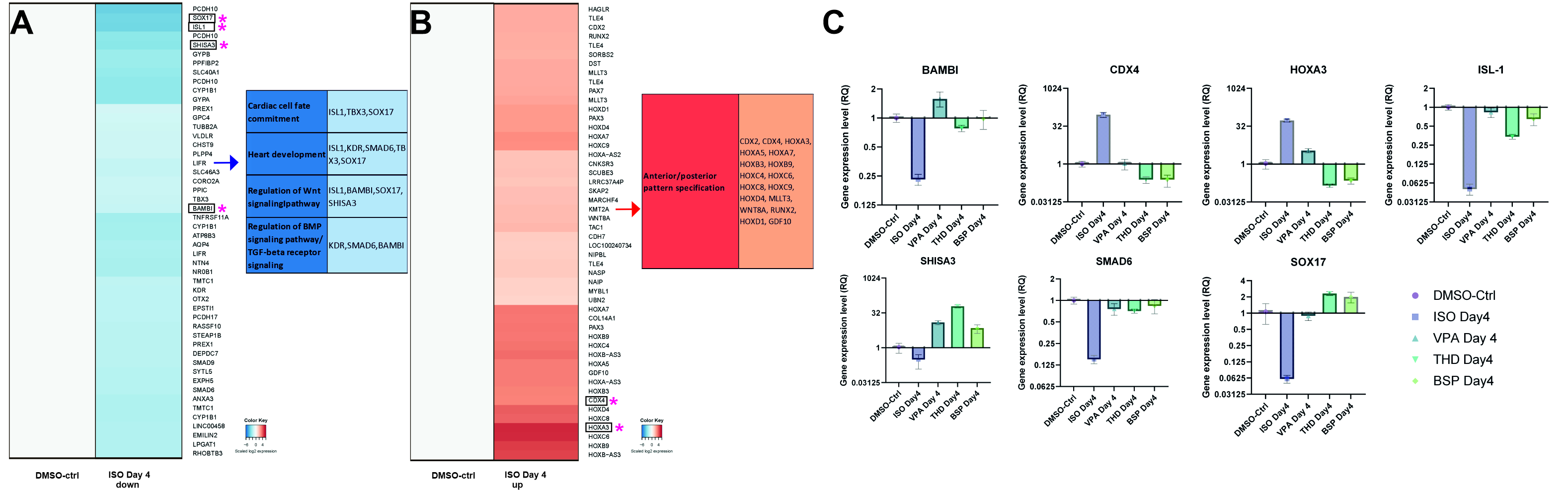

Supplement: Supplementary file 4 — Figure S4 [file 41420_2023_1616_MOESM4_ESM.jpg]

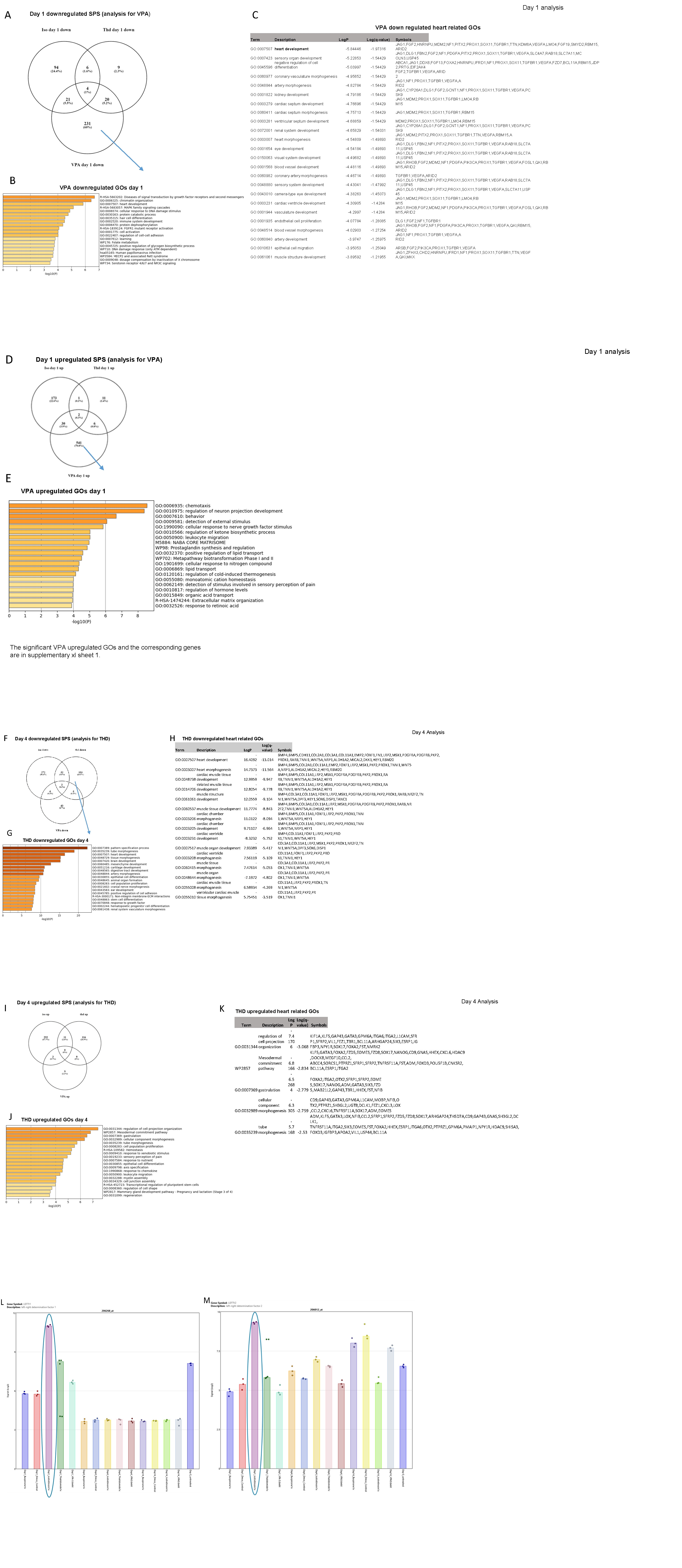

Supplement: Supplementary file 5 — Figure S5 [file 41420_2023_1616_MOESM5_ESM.jpg]

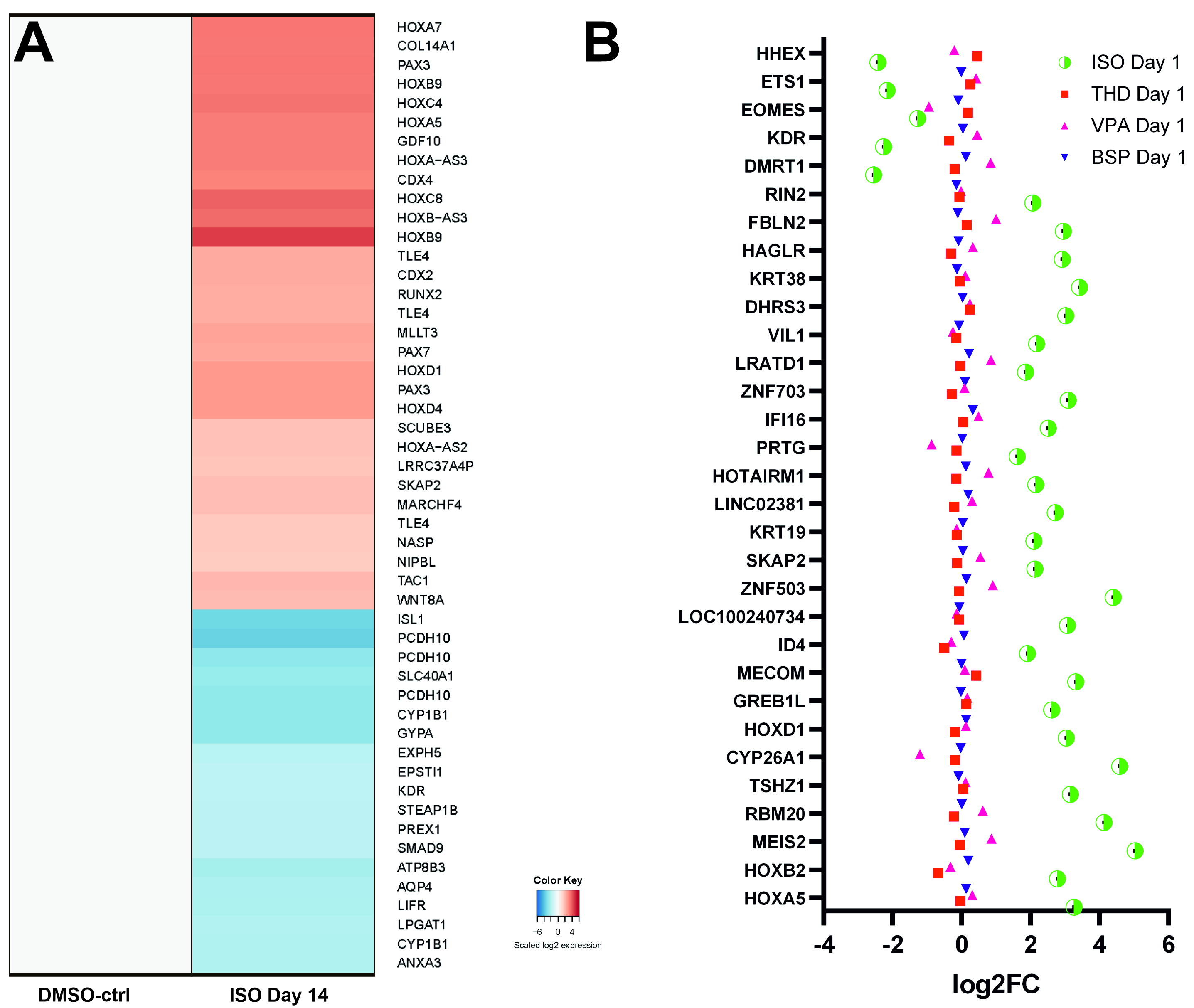

Supplement: Supplementary file 6 — Figure S6 [file 41420_2023_1616_MOESM6_ESM.jpg]
